# A study of the initial adhesive force of cells on silk fibroin-based materials using micropipette aspiration

**DOI:** 10.1093/rb/rby004

**Published:** 2018-03-15

**Authors:** Xiaojie Lian, Shichao Liu, Liming Liu, Rui Xu, Miaomiao Du, Song Wang, Hesun Zhu, Qiang Lu, Quanyou Zhang, Yali Wu, Di Huang, Yan Wei

**Affiliations:** 1 Department of Biomedical Engineering, Research Center for Nano-Biomaterials and Regenerative Medicine, College of Mechanics, Taiyuan University of Technology, Taiyuan 030024, China; 2 Shanxi Key Laboratory of Material Strength and Structural Impact, Institute of Applied Mechanics and Biomedical Engineering, Taiyuan University of Technology, Taiyuan 030024, China; 3 Research Center of Materials Science, School of Materials Science and Engineering, Beijing Institute of Technology, Beijing 100081, China; 4 National Engineering Laboratory for Modern Silk, College of Textile and Clothing Engineering, Soochow University, Suzhou 215123, China

**Keywords:** silk-based materials, cell initial adhesion, micropipette, uniform cells in round shape

## Abstract

With the development of biomaterials, more attention is paid to the adhesion characteristics between cells and materials. It is necessary to study the adhesive force with a suitable method. Silk fibroin (SF) is widely investigated in biomedical application due to its novel biocompatibility and mechanical properties. In this article, the micropipette aspiration method and measurement pattern of uniform cells in round shape (UCR) was used to study the initial adhesive force of three types of cells on pure silk fibroin films (SFFs). We also compared the adhesive forces of modified SFFs with that of pure SFFs. The results of adhesive force in the initial adhesive stage were in concordance with the results of MTT assay and microscope observation, which were confirmed by the above three cell lines and four kinds of SFFs. The results indicated UCR was an efficient and quantitative measurement pattern in initial adhesion stage. This article also provides a useful method in identifying initial cell-materials interactions.

## Introduction

Cytocompatibility is one of the most important aspects of biocompatibility of biomaterials [1–3]. Cell-substrate adhesion affects follow-up cellular functions, such as migration, proliferation and differentiation [4, 5]. Thus, it is necessary to study the adhesion characteristics with a suitable method. Micropipette aspiration technique was utilized to investigate the adhesive force of a single cell quantitatively. However, some of these studies have technical difficulties, such as the need for protein scaffolding of the cells to avoid membrane rupture when they are pulled by a nano- or micro-needle [6, 7]. The first step of cell-surface adhesion was considered as the initial contact of cells with materials, in which cells were in round shape but no cytoskeletal change yet. The measurement pattern of uniform cells in round shape (UCR) was already used in evaluating the endothelial cell adhesive properties on various silk fibroin (SF)-based materials in our previous study [4, 5]. This method shows high reproducibility and sensibility in evaluating the cellular affinity quantitatively with a small amount of uniform cells in earlier adhesion stage. However, the mechanism of how the adhesive force of cells on silk-based materials does changes, and whether the performance would indicate the follow-up cell activities have not been clarified yet. The purpose of this study is first to investigate the adhesive force of fibroblasts (NIH-3T3), osteoblasts and endothelial cells (ECV304) on pure silk fibroin films (SFFs) and tissue culture polystyrene dishes (TCPS) using the UCR measurement pattern.

Some traditional Chinese medicines have been used in China for a long time. Ejiao (E) has been widely used to treat gynecologic and chronic diseases [8]. Fucoidan (FC), a sulfated polysaccharide, has shown various functional and biological properties, such as antioxidant and anti-viral activities [9, 10]. Danshensu (DS) has been extensively used for the treatment of angina pectoris, myocardial infarction and stroke [11]. In this study three kinds of the afformentioned Chinese herb given above and fibronectin (FN), which is involved in cell proliferation, migration, differentiation and survival as a major extracellular matrix component, were mixed to SF to prepare SF modified films [12]. E, FC, DS and FN modified SFFs were prepared, on which the cellular adhesive force were compared with pure silk material using UCR measurement pattern by micropipette aspiration in this study. Later proliferation and cell morphology was observed by optical microscope and tested by MTT array, respectively [13, 14].

The aim of this study was to build and verify an efficient and quantitative measurement pattern of UCR with micropipette aspiration and find the relation between initial adhesive force and later proliferation for cells on SFF substrates in initial adhesion stage. The research would be useful to seek useful scaffold or substrate with excellent cytocompatibility by investigating initial adhesive performances of cells on silk-based substrates.

## Materials and methods

### Cell lines

ECV-304 human umbilical vein endothelial cell and NIH-3T3 mouse embryonic fibroblasts were obtained from the Institute of Basic Medical Sciences, Chinese Academy of Medical Sciences. Osteoblasts (MC-3T3) were kindly provided by School of Life Science, Beijing institute of technology, China. They were all routinely cultured in our lab with Dulbecco’s Modified Eagle’s Medium-High Glucose (Invitrogen, America) in a humidified air with 5% CO_2_ at 37°C using standard cell culture techniques. The culture was added with 10% (v/v) bovine calf serum (New Probe, China), 100 μg/ml penicillin as well as 100 μg/ml streptomycin.

### Preparation of SFFs

Silk (Yi Xian Raw Silk Factory, China) was heated in 0.5 wt % (w/w) Na_2_CO_3_ aqueous solution at 100°C for an hour and rinsed with deionized water three times to remove the outer sericin. The degummed SF was dissolved in the solution at 80°C for 30 min with a composition of CaCl_2_, H_2_O and C_2_H_5_OH (molar ratio, 1:8:2) [15, 16]. The SF solution was obtained after dialysis against deionized water for 3 days, which was filtered and then diluted to 4 mg/ml for use. For micropipette aspiration measurements, SF solutions were formed films on TCPS, while were prepared membranes for MTT test, on 96-well plates at 60°C for 24 h. All silk films were soaked in 75% (v/v) alcohol for half an hour, followed by sterilization using ultraviolet ray for 2 h. TCPS was also used as a control to be measured.

E was supplied by Shandong Dong-E-E-Jiao Co., Ltd (China). FC from Fucus vesiculosus was purchased from sigma-aldrich (America). And DS sodium salt was purchased from sigma-aldrich (America) in this study. Four mg/ml E was added to 4 mg/ml SF solution with the 5 wt% ratio and dried at 60°C to obtain SFF/E film. The conditions for preparing SFF/FC and SFF/DS were similar. Different concentrations of FN (Sigma, China) were coated on the surface of SFFs, incubated at R.T. for 2 h, rinsed with PBS two times and dried. Mixtures of SFF with 10, 50 and 600 μg/ml of FN were each denoted as SFF/FN-10, SFF/FN-50 and SFF/FN-600, respectively.

### One-by-one test of adhesive force between cell and material using micropipette aspiration

**Figure 1.**
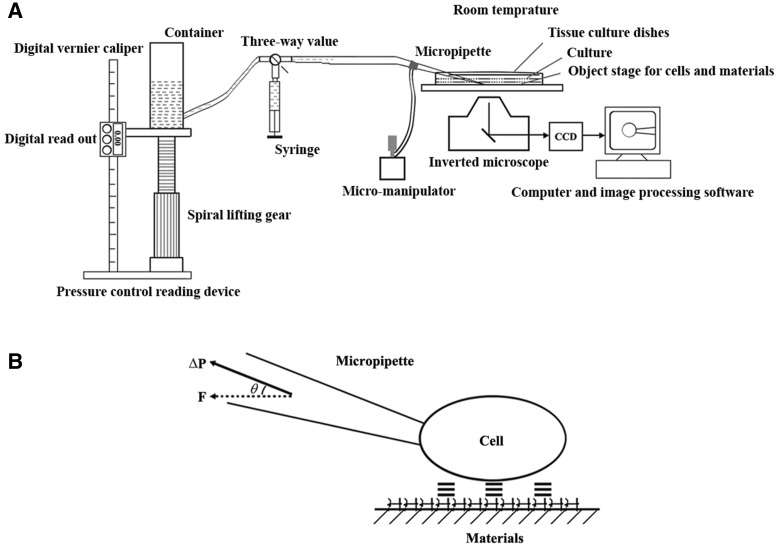
(**A**) The schematic diagram of micropipette system. **(B)** Schematic description of detachment force model of cell from substrate

Figure 1A illustrated the schematic diagram of micropipette aspiration system, which was used in the measurement of adhesive force as former study [4]. Briefly, it makes up of a pressure control reading device, a micro-manipulator (ONO-301D, Narishige, Japan), an inverted microscope (IX71, Olympus, Japan), a charge-coupled device (CCD) camera (MP3.3-RTV-CLR-10, Olympus, Japan), a computer and image processing software (Image-Pro Express Ver 5.1 w, Olympus, Japan) and a micropipette. Figure 1B showed the schematic description of adhesive force model of cell from substrate. The critical adhesive force was noted as *F*, which is equal to π*r*^2^Δ*p*cosθ[17], where r is the inner radius of a micropipette (μm), Δ*p* is the critical vacuum pressure caused by hydrostatic column (Pa) and θ is the angle between micropipette and the surface of films. In this article, θ was required to be <10°, so cosθ in the formula is approximately equal to one. Additionally, Δ*p* was calculated as Δ*p* = ρgh, so, 1 mm H_2_O is equivalent to 9.806 65 Pa. Therefore, the formula could be simplified as *F* = 3.079 *hr*^2^ ×1 0 ^−11^ N.

The cells were seeded at 37°C in incubator for certain time, after that the floating cells were thoroughly rinsed, and then tested one-by-one with micropipette aspiration in room temperature at 25°C.

### MTT assay

Cell growth behavior can be evaluated by MTT (Genview, China) test, which was a common method of the relative viability of the cells cultured on substrates [18, 19]. Cells (7 × 10^3^–7 × 10^4^ cells/cm^2^) were incubating in 96-well plates coated with SFFs for certain time. The MTT solution of 5 mg/ml was prepared by dissolving MTT in sterile filtered PBS, then 20 μl of which was added to each sample hole at 37°C. After 4 h of incubation, the supernatant was removed by 150 μl DMSO solution to dissolve the formazan crystal. The solution was shaken homogeneously for 10 min. The optical densities were recorded by a microplate reader (680, Bio-Rad, USA) at 490 nm. For reducing the influences of light absorbance through films, all components except cells were added to each substrate-well and were used as the control sample. The cell photos were taken by microscope regularly.

### Statistical analysis

All the data were statistically analyzed using SPSS software and expressed as the mean ± SD. The t-test was performed and *P* < 0.05 was considered statistically significant.

## Results

### Comparison of the cell adhesive forces of SFF and TCPS in different force intervals with three kinds of cells

To compare the cell adhesive forces of SFF and TCPS, various kinds of cells were employed and incubated on surfaces of SFF and TCPS for different times at 37°C then tested at 25°C. The culture time might be different depending on different cell types and cell batches, which should make sure a few round shape cells adhering on surfaces in all groups. Table 1 showed cell adhesive forces on the two materials with three types of cells in two periods of testing time.

**Table 1:** Cell adhesion condition on SFF and TCPS with three types of cells

Test	Cell type	Culture time(min)	Testing time(min)
1	NIH-3T3	15	0–90
			90–150
2	Osteoblast	15	0–90
			90–150
3	ECV304	60	0–90
			90–150

The number of cells to be measured in all groups in Table 1 is about 15–20.

The culture temperature is 37°C and then the testing temperature is 25°C.

**Figure 2.**
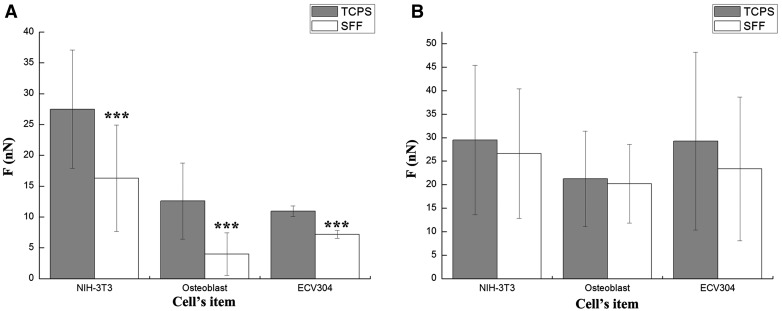
The comparison of cell adhesive forces between SFF and TCPS at different adhesion stage of (**A**) the initial stage between 0 and 90 min; (**B**) the subsequent stage between 90 and 150 min

Figure 2 made the comparison of cell adhesive forces between SFF and TCPS in different adhesion stage divided by Table 1. Figure 2A shows the initial stage between 0 and 90 min and Fig. 2B shows the subsequent stage between 90 and 150 min. In tests, cells adhered onto both two materials, and the cell adhesive force on TCPS was greater than that on SFF, moreover, there was a significant difference between the cell adhesive forces of two materials (*P* < 0.001) in 90-min test for three types of cells. In the subsequent testing time of 90–150 min, cells more strongly adhered onto the two materials, but there was no significant difference between the cell adhesive forces of the two materials (*P* > 0.05) for all group. In other words, the results also indicated that there might exist initial adhesion stage in force, specifically when the initial adhesive forces of NIH-3T3, Osteoblasts and ECV304 was (16.1 ± 8.5), (4 ± 3.4) and (7.1 ± 0.7) nN on SFF respectively, there was a significant difference between the cell adhesive forces of TCPS and SFF. In follow-up stage, when the adhesive force of the three cells was (26.5 ± 13.7), (20.1 ± 8.3) and (23.3 ± 15.1) nN on SFF respectively, there was no significant difference between the cell adhesive forces of the two materials. The results also have suggested that the cell adhesive force in the initial stage could behave significantly different on diverse materials, which became less obviously in testing time for 90–150 min.

### Influence of culture time on cell adhesive force on SFF

In order to explain the phenomenon that comparison of the cell detachment forces on TCPS and SFF was not consistent in different adhesion stage, the influences of culture time and temperature on the cell detachment force of SFF were studied.

**Figure 3.**
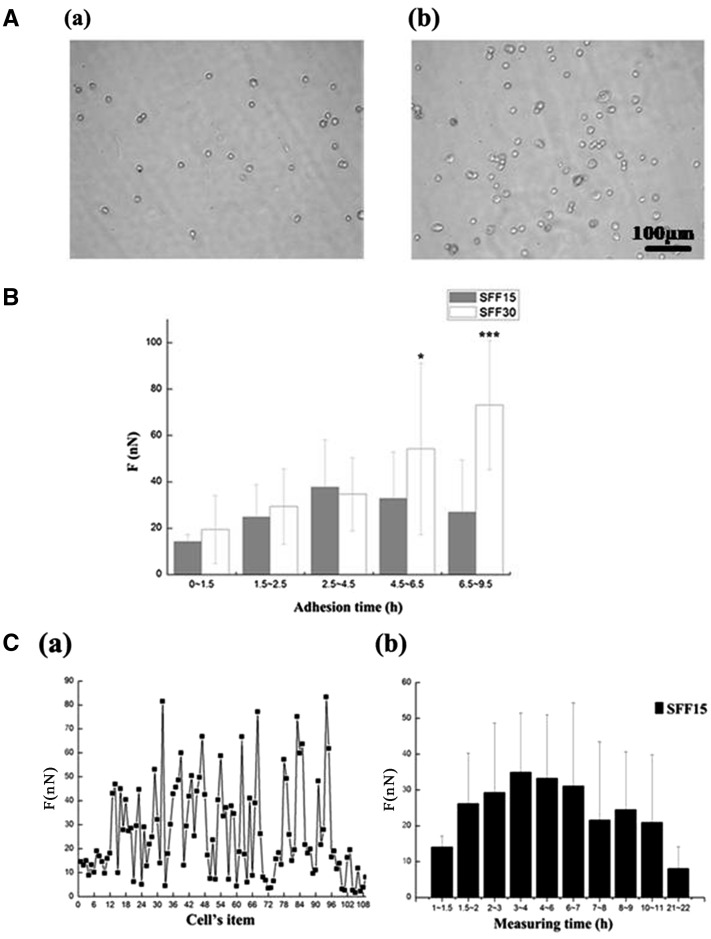
(**A**) Comparison of NIH-3T3 density between different incubation time at 37°C (**a**) 15 min (**b**) 30 min. (**B**) Comparison of adhesive force between SFF15 and SFF30 in 0–9.5 h at RT. **(C)** The change curve (**a**) and column chart (**b**) of NIH-3T3 adhesive force cultured on SFF for 15 min at 37°C and testing at RT. between 0 and 22 h

Figure 3 showed a comparison of adhesive force for culture time between 15 and 30 min on SFFs, which labeled as SFF15 and SFF30 separately. In the experiment, the same batch of NIH-3T3 was cultured on SFF at 37°C for 15 and 30 min separately, and then the floating cells were thoroughly rinsed. The density of NIH-3T3 shown in Fig. 3A increased with the increase of culture time. The adherent cells in round shape as the standard cell line were chosen to study the cell adhesive force on different materials using the UCR method. NIH-3T3s had the features of the ‘standard cell’ after 15-min incubation on SFF, but began to spread and deform on the substrate after 30 min individually. The adhesion force of a single NIH-3T3 on SFF surface at 25°C out of incubator was further tracked in Fig. 3B. SFF15 increased within 2.5 h, reaching its maximum force between 2.5 and 4.5 h and decreased gradually between 4.5 and 9.5 h. SFF30 increased between 0 and 9.5 h, but had not reached its maximum before the end of test, that means the maximum of SFF30 may appear after 9.5 h. Thus, the fluctuation range of SFF30 is much larger than SFF15. It illustrated that shorter incubation time on SFF may resulted in lower adhesive force that would shorten testing period for the UCR method. The influence of testing time at RT of 25°C on NIH-3T3 adhesion strength was studied and explained in Fig. 3C. The adhesive force of NIH-3T3 was measured at 25°C after culturing on SFF for 15 min at 37°C. More than 100 NIH-3T3s were tested during 22 h. Cells showed cytoplasm shrank and died between 2 1 and 22 h during the test. Figure 3A was a chart of detachment force of 108 cells, which were tested one-by-one. The mean data and variance of adhesive force in certain period of measuring time was also calculated in Fig. 3B. It illustrated that the adhesive force data of the 12 cells were concentrated, lower and scattering between 0 and 1.5 h, then increased between 1.5 and 4 h and decreased gradually between 4 and 11 h. The results indicated that the initial adhesion time at RT could be chosen to test by UCR method before the period of NIH-3T3 adhesive force on SFF increasing with large fluctuations or decreasing because of death.

### Comparison between SFF and TCPS with three kinds of cells

**Figure 4.**
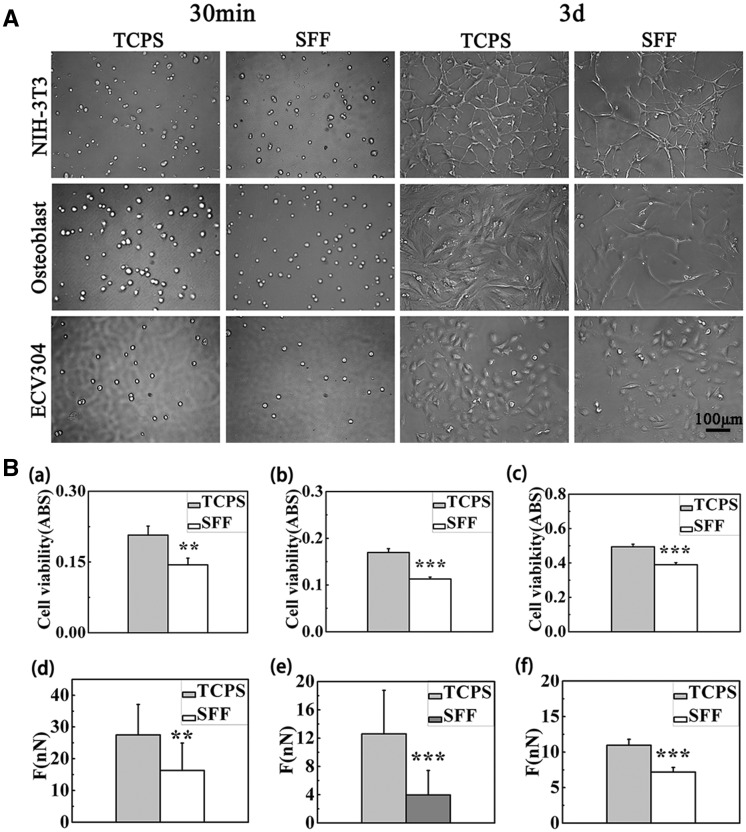
(**A**) The optical microscope images of cell morphology on TCPS and SFF. (**B**) Cell activity on TCPS and SFF measured by MTT assay for ((**a**) NIH-3T3, (**b**) osteoblast and (**c**) ECV304) and adhesive force measured for ((**d**) NIH-3T3, (**e**) osteoblast and (**f**) ECV304)

Figure 4A showed the cell proliferation morphology on SFF and TCPS by optical microscope at different stage. Cells were in round shape after incubation of 30 min on both materials, and then all types of cells began to protrude pseudopodium obviously after 3 days. Nevertheless the cell morphology on TCPS was spread better than on SFF. The surface of TCPS was overgrown with a single cell layer and the cell density on SFF was obviously less than that of TCPS. Figure 4B(a–c) illustrated the comparison of three types of cell relative proliferation between TCPS and SFF for 3 days. The absorbance value of NIH-3T3 on TCPS was greater than on SFF and there was a significant difference between them, which suggested that the ability of cell adhesion and proliferation on TCPS was better than SFF. Figure 4B(d–f) indicated that the larger cell initial adhesion force on TCPS than that on SFF with the UCR method. Above all, the result of initial adhesive force was concordance with the result of MTT assay and microscope photos.

### Comparison between NIH-3T3 adhesive forces of SFF and silk-based materials

**Figure 5.**
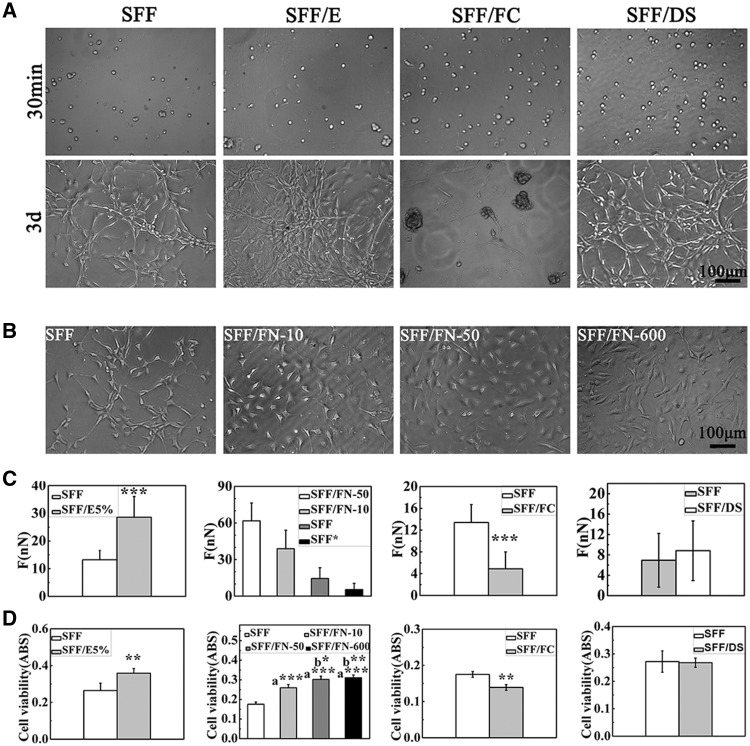
(**A**) The optical microscope images of NIH-3T3 morphology on SFF, SFF/E, SFF/FC, SFF/DS; and **(B)** SFF/FN. **(C)** Comparison between NIH-3T3 adhesive force on silk-based materials and **(D)** Cell activity on silk-based materials measured by MTT assay (a*** denotes *P* < 0.001 (compared with SFF); b** denotes *P* < 0.01 (compared with SFF/FN-10); b* denotes *P* < 0.05 (compared with SFF/FN-10))

Figure 5A showed the cell morphology and density of cells cultured with SFF, SFF/E, SFF/FC and SFF/DS. There was no obvious difference between them for 30 min in optical microscope photos. In 3 days, the surface of SFF/E was overgrown with single layer and the cell density was obviously more than that of SFF. However, the cells gathered to form a larger mass on the surface of SFF/FC. NIH-3T3 on SFF/DS grew to form a single layer and the cell density obviously increased. Figure 5B showed the surface morphology of NIH-3T3 on SFF and SFF/FN for 1 day through optical microscope. On the surfaces of SFF/FN, NIH-3T3 was plumper than on SFF in morphology. NIH-3T3 adhesive force between SFF and silk-based materials was compared in Fig. 5C. SFF/E5%, SFF/FC, SFF/DS and SFF/FN were all incubated at 37°C for 15 min, and then tested at R.T for 25°C between 0 and 30 min. Regarding the adhesive forces of NIH-3T3, SFF/E > SFF > SFF/FC, SFF/FN-50 > SFF/FN-10 > SFF > SFF* (without serum). There was a significant difference between the NIH-3T3 adhesive forces of any two groups except for SFF and SFF/DS. Figure 5D showed the cell activity on silk-based materials by MTT. Regarding the light absorption value of NIH-3T3, SFF/E > SFF > SFF/FC, SFF/FN-50 > SFF/FN-10 > SFF. There was also no significant difference between the NIH-3T3 light absorption value of SFF and SFF/DS, which in accordance with the result of adhesive force by UCR method. The light absorption value increased as the concentration of FN increased between 0 and 50 μg/ml and there was no significant difference between 5 0 and 600 μg/ml. A total of 600 μg/ml was the saturation point for protein absorption. The result accorded with literature [20].

## Discussion

In this study, the measurement pattern of UCR was used to measure quantitatively one-by-one with only a few round shape cells in certain time to distinguish the different adhesive force between substrates. The size of force displayed narrow distribution in initial adhesive stage, while the data fluctuated strongly, if cells attached or spread well on materials in follow-up stage that would need larger force and more time to suck cells or lead to rupture. The initial adhesion characteristics of NIH-3T3, osteoblasts and ECV304 on SFF and TCPS were investigated using the improved UCR measurement pattern for obtaining the initial adhesive force. It was concluded that there was a significant difference between cell adhesive forces of SFF and TCPS in the initial adhesion stage; however, there was no significant difference between cell adhesive forces of TCPS and SFF in follow-up stage, which is similar to results of proliferation and viability by microscope and MTT assay. Therefore, the initial adhesive force could be tested and compared efficiently and reproducibly with UCR for the adhesion between kinds of cells and biomedical substrates.

The influences of incubation time as well as testing time on cell detachment force on SFF for the same cell recovery batch of 3T3 were discussed to explain the different result comparison of cell detachment forces on TCPS and SFF at different cell adhesion stages. Comparing to the culture atmosphere at 37°C in incubator, there may exist in two cell states for the different period at 25°C out of it. First, the cells were still alive for earlier time, which grew and spread on SFF continuously. And the cell adhesive force increased gradually in this period. Second, the cell adhesive force would decrease because of condition changes such as temperature reduction as well as variation of the PH value of the medium and so on over a long period of time. It indicated that cells with relatively lower and more stable adhesive force on substrates could be feasible to test by UCR method in the initial adhesion time, when cells were less affected by environmental impact.

The difference of cell adhesion characteristic on diverse substrates decide subsequent cell migration, cell proliferation as well as the formation of tissue and organ, which is therefore essential to study initial adhesion for designing biomaterials in tissue engineering field. Adhesive force occurs between cells and their supporting matrix as well as between cells [21, 22]; however, only the former is the subject of our study. Different modified SFFs were used as biomedical materials, on which cells would exhibit diverse biological performance. For the purpose of distinguishing adhesion forces of various cell-silk based materials, four kinds of modified silk films with similar formation process were employed in evaluating the UCR method in this study. The results of initial adhesive force for four silk-based materials were also confirmed by results of MTT assay and microscope photos, which indicated the versatility and reliability by the UCR method.

In other words, the initial adhesion forces might predict the relative merits of subsequent change of cellular behavior such as adhesion and proliferation between different cell and substrate. The biological difference between materials and cells might already act out in the initial phase, when force could easily be measured and compared by mechanical method using micropipette aspiration. However, the adhesive forces became larger and disperse as the increase of culture time and make it difficult to test, when cell cytoskeleton changed.

## Conclusions

In this article, cell adhesive force of the UCR measurement pattern with micropipette aspiration was studied. A few uniform round cells were tested at initial adhesive stage. Cell adhesion force was relative steady at initial stage, while was shown wide distribution at follow-up stage, which was affected by cell incubation time and testing time for SFF. Furthermore, initial adhesion stage was considered as a boundary for cell force on SFF substrates, in which the results of force for cell-material were identical with results of proliferation and viability by microscope observation and MTT test. The effectiveness as well as reproducibility of measurement pattern was confirmed by three cell lines and four kinds of SFF modifying with Chinese herb in initial adhesion time. The results also indicated that initial adhesive force by UCR measurement pattern would be helpful and efficient to distinguish cell affinity in earlier adhesion time between different scaffolds and provide the useful and quick method to choose and design the surface properties of a novel scaffold for tissue engineering in future work.

## Funding

This work was supported by the National Natural Science Foundation of China (NSFC, no. 51502192, 11502158, 31501212 and 51503140), Scientific and Technological Innovation Programs of Higher Education Institutions in Shanxi (STIP, no. 2016142), National Basic Research Program of China (973 project, 2005CB623906). Natural Science Foundation for Young Scientists of Shanxi Province (no. 2014021039-6 and 201601D021127), the Qualified Personnel Foundation of Taiyuan University of Technology (QPFT, no. tyut-rc201270a), the Youth Foundation of Taiyuan University of Technology (no. 1205-04020102, 2013Z020 and 2014TD066) and the Technical Services Project of Taiyuan University of Technology (no. 143230043-J).

*Conflict of interest statement*. None declared.
